# Influence of Electroless Nickel—DLC (Diamond-like Carbon) Multilayer Coating on the Mechanical Performance of the Heat-Treated AlSi10Mg Alloy Produced by Powder Bed Fusion-Laser Beam

**DOI:** 10.3390/ma16093313

**Published:** 2023-04-23

**Authors:** Gianluca Di Egidio, Carla Martini, Lorella Ceschini, Alessandro Morri

**Affiliations:** Department of Industrial Engineering (DIN), Alma Mater Studiorum, University of Bologna, Viale del Risorgimento 4, 40136 Bologna, Italy; gianluca.diegidio2@unibo.it (G.D.E.); lorella.ceschini@unibo.it (L.C.); alessandro.morri4@unibo.it (A.M.)

**Keywords:** powder bed fusion-laser beam (PBF-LB), AlSi10Mg alloy, tensile test, fatigue test, Fractographical analysis, diamond-like carbon (DLC), electroless Ni-P coating, heat treatment

## Abstract

This study characterizes the mechanical performance of the AlSi10Mg alloy produced by powder bed fusion-laser beam (PBF-LB) subjected to two combined cycles consisting of multilayer coating deposition (electroless nickel (Ni-P) + diamond-like carbon (DLC)) and heat treatment. In particular, the DLC deposition phase replaces the artificial aging step in the T5 and T6 heat treatments, obtaining the following post-production cycles: (i) Ni-P + DLC deposition and (ii) rapid solution (SHTR) (10 min at 510 °C) before Ni-P + DLC deposition. Microstructural characterization shows no appreciable modifications in the morphology and dimensions of the hard Si-rich phase of the eutectic network and secondary spheroidal Si phase. However, overaging phenomena induced by DLC coating deposition and differences in elastic-plastic properties between the multilayer coating and the PBF-LB AlSi10Mg substrate lead to a reduction in tensile strength by up to 31% and a significant decrease in ductility by up to 58%. In contrast, higher resistance to crack opening thanks to improved surface hardness and residual compressive stresses of the coating and reduced defect sensitivity of the substrate increase the fatigue resistance by 54% in T5-coated alloy and 24% in T6R-coated alloy. Moreover, the coating remains well adherent to the substrate during fatigue testing, not becoming a source of fatigue cracks.

## 1. Introduction

Aluminum-silicon (Al-Si) alloys are used for a wide range of engineering applications (from the transportation to the packaging sector, comprising more than 90% of total aluminum castings [1]). These alloys also attract remarkable research interest thanks to the possibility of using additive manufacturing processes for component production [2,3,4]. However, the tribological behavior of Al-Si alloys proved to be unsatisfactory in many applications involving sliding motion, owing to their low adhesion and abrasion resistance [5]. Therefore, to improve the tribological behavior of these alloys, surface modification techniques, including physical vapor deposition (PVD), plating/electroplating, anodizing, thermal spraying, and laser-based surface treatments, can be used as it is well documented [6]. Among these techniques, electroless nickel (Ni-P) plating has been selected for depositing a load-bearing interlayer on AlSi10Mg produced by powder bed fusion-laser beam (PBF-LB) before applying a Diamond-like Carbon (DLC) topcoat, with the final aim of improving both tribological behavior and fatigue behavior. The Ni-P interlayer was selected because of the high throwing power of electroless deposition, which makes it an optimal solution when the geometry of components is complex, as is often the case for additively manufactured parts. For this reason, surface engineering methods based on microstructural modification, such as friction stir processing [7], which is known to improve surface hardness but requires simple geometries, or laser shot peening, which is a well-known method to improve fatigue behavior [8] but which suffers from limitations on the geometry and the dimension of the component, were not taken into account.

Ni-P plating has drawn attention over a decade among the surface engineering methods used to protect structural materials such as steel and Al alloys by increasing corrosion and wear resistance [9,10]. In particular, the deposition process is relatively simple and inexpensive; it is based on an autocatalytic redox reaction in which Ni^2+^ ions are reduced by sodium hypophosphite (NaH_2_PO_2_). The reducing agent (P) is incorporated into the growing layer, thus improving the pristine metal (Ni) properties by alloying them with P [11]. This factor makes the P content one of the main parameters influencing the properties of the Ni-P coating. For instance, a medium-high P content (>8 wt%) improves corrosion resistance and introduces functional compressive stresses into the Ni-P coating thanks to deposition conditions [12,13]; in particular, the higher the P content in the deposit, the more likely the stress will be compressive [14]. However, Ni-P deposits with the same P content but deposited onto different substrates show significantly different compressive stress values due to the different expansion coefficient values of the substrate. Generally, in Al alloys, a P content higher than 6 wt% is sufficient to introduce residual compressive stresses in the Ni-P coating [14,15].

Ni-P-coated structural components may undergo static or cyclic loading, which can induce component fracture. In particular, several investigations into the effect of high P content in Ni-P coatings on the mechanical properties of steels and Al alloys have focused on the factors affecting the performance of the Ni-P-coated parts: substrate-to-coating-strength ratio, coating thickness, internal residual stresses, and heat treatment [13,16,17,18,19,20,21,22]. In recent work, Puchi-Cabrera et al. [18] showed that the Ni-18%P coating could significantly improve the fatigue and corrosion-fatigue performance of the 7075-T6 alloy. According to the authors, the Ni-P deposit shows excellent adhesion to the substrate even when the system is subjected to tensile stresses exceeding the yield strength (YS). Furthermore, the compressive nature of the residual stresses in the Ni-P interlayer contributes to improving the fatigue performance of the coated alloy. However, Rahmat et al. [20] evaluated a significant loss in ductility for the coated 7075-T6 alloy (54% less than the uncoated Al alloy) due to the brittleness of the Ni-P coating. In particular, the coating cracked under loading and transferred the stored energy to the substrate, leading to a fast failure, even though a 6% increase in YS and ultimate tensile strength (UTS) was observed in the Ni-P coated specimens. As described by Kumar et al. [21], this effect strongly depends on the thickness of the Ni-P coating deposited on the Al substrate: as the thickness increases, the strength properties increase and the ductility decreases. The increase in fatigue properties induced by the Ni-P coating does not occur only in the 7075-T6 alloy; as Lonyuk et al. [13] described, the deposition of a Ni-13.2%P coating on an AA 2618 substrate significantly increased the fatigue life of the specimens.

DLC coatings exhibit high hardness and good wear resistance and have been widely used in the past few years on light alloys, such as Al-based and Ti-based alloys, to reduce contact friction in energy, transportation, and medical applications [23]. Several types of DLC films can be deposited onto metal substrates, including amorphous carbon (a-C), tetrahedral amorphous carbon (ta-C), hydrogenated amorphous carbon (a-C:H), and hydrogenated tetrahedral amorphous carbon (ta-C:H), and they are selected on the basis of specific applications [24]. An important characteristic of the growth mechanism of DLC coatings is the generation of compressive residual stresses (of the order of 1 GPa [25]), which can improve the fatigue properties of the Al substrates. However, as Baragetti et al. [26] reported, the direct application of DLC coating on Al alloys could reduce the fatigue response of the component; in fact, even though DLC films have excellent tribological properties (i.e., low friction coefficient combined with high wear resistance), direct deposition causes the formation of cracks in service in the coating, linked to the low load-bearing capacity of the substrate.

A multilayer solution can solve the problems related to the direct application of DLC on the Al substrate; it involves the sequential application of two (or more) surface modification processes to produce combined properties not achievable with a single surface treatment, possibly avoiding individual drawbacks. In particular, the Ni-P + DLC multilayer coating can be an optimal solution to increase the tribological and mechanical performance of the Al alloys: the hard interlayer (Ni-P) enhances the load-bearing capacity of the substrate, improving the top-coating (DLC) adhesion, which in turn reduces the wear rate and the coefficient of friction in the system.

However, even though the advantages of multilayer coatings on the tribological properties of Al alloys are widely documented [11,23,27,28,29,30], there are no studies on the combined tensile and fatigue performance of Ni-P + DLC multilayers. Given the high mechanical stress to which Al-based structural components are subjected, it is essential to know how the mechanical properties of parts change in coated and uncoated conditions.

In light of the above, this paper focuses on the effect of a Ni-9%P + DLC (hydrogenated amorphous carbon, a-C:H) multilayer coating on the microstructure and mechanical behavior of the PBF-LB-produced AlSi10Mg alloy, which is characterized by good thermal conductivity and weldability, low solidification shrinkage and cracking, and a high strength-to-weight ratio. These features enable the production of high-performance and lightweight structural components, reducing fuel consumption and CO_2_ emissions in automotive and aerospace applications [31,32].

In particular, the study evaluates the tensile and fatigue properties of the alloy subjected to two innovative integrated deposition and heat-treatment cycles: (i) T5-like heat treatment, i.e., Ni-P + DLC deposition on as-built (AB) alloy and (ii) T6R-like heat treatment, i.e., rapid solution (SHTR) at 510 °C for 10 min [33], followed by Ni-P + DLC deposition. In these cycles, the final DLC deposition (carried out at about 180 °C) replaces artificial aging (AA).

## 2. Materials and Methods

### 2.1. Sample Production and Post-Processing Cycles

**Figure 1:**
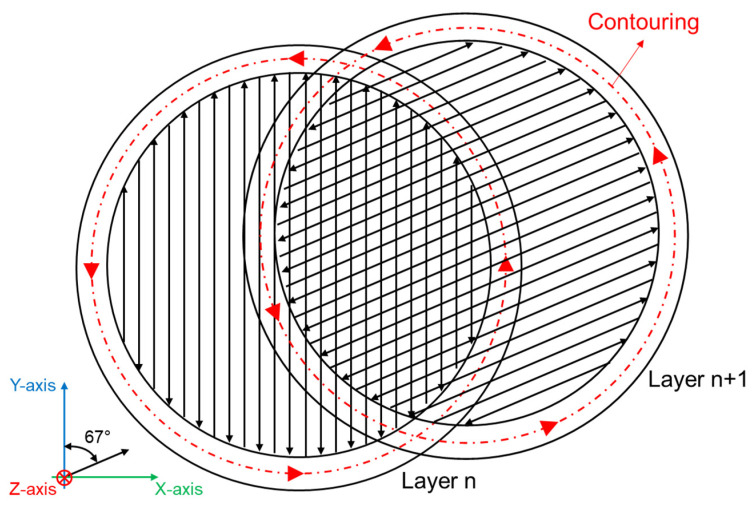
Scan strategy adopted to make PBF-LB AlSi10Mg samples: bidirectional stripes and 67° anticlockwise rotation on each successive layer, and remelted contour zone strategy at the end of each scanning.

Tensile and fatigue specimens were printed by using a SLM500 system (SLM Solutions, Lübeck, Germany), which is characterized by a 150 °C heated-platform temperature to reduce the thermal gradient during printing, and filled with a high-purity Ar gas to diminish the O_2_ level in the building chamber to 0.2 vol.%. Specimens are characterized by the longitudinal axis parallel to the building direction to reduce industrial costs [34] and consider the worst-case scenario for mechanical properties [35]. Process parameters are detailed in Table 1, and the scheme of the scan strategy is reported in Figure 1. More information about the powder’s physical and chemical features can be found in previous work [33].

**Figure 2:**
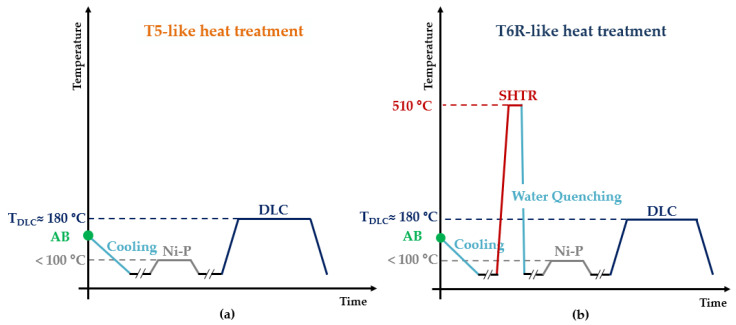
Integrated post-processing cycle: T5-like heat treatment (AB + Ni-P + DLC) (**a**); T6R-like heat treatment (AB + SHTR + Ni-P + DLC) (**b**).

This study analyzes integrated coating and heat-treatment cycles for the PBF-LB AlSi10Mg alloy, in which the DLC deposition phase substitutes the AA step. In fact, during the DLC deposition, performed at about 180 °C for 4–5 h [36], the heat-treated alloys undergo precipitation hardening and residual stress relief. Figure 2 reports the schemes of the two integrated cycles: T5-like (T5-C), which maximizes the mechanical strength and reduces residual stresses, and T6R-like (T6R-C), which increases the balance of the tensile properties, reduces residual stresses and improves fatigue strength [33,37].

**Figure 3:**
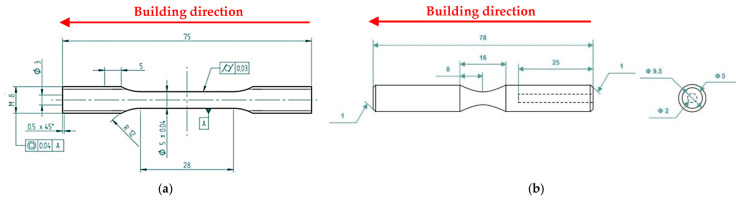
Tensile (**a**) and fatigue (**b**) specimen geometry. Longitudinal axis of the samples is parallel to the building direction.

Tensile and fatigue specimens (Figure 3) were machined from AB specimens, as described in [33,37]. T5-C specimens were subjected to Ni-P and DLC deposition and then tested, while the T6R-C ones were solubilized before the deposition of Ni-P and DLC. SHTR (10 min at 510 °C) was conducted in an electric furnace with a temperature control of ±5 °C. Two K-type thermocouples were placed next to the specimens to check temperature uniformity during furnace holding time.

Medium Ni-P coating (9 wt% P) was deposited in an industrial facility at temperatures lower than 100 °C, providing a negligible effect on substrate microstructure given the printing conditions (Table 1). For the T6R-C specimens, Ni-P deposition occurred after the SHTR step to avoid the formation of thin Ni oxide and consequent problems during the DLC coating deposition, carried out by Arc-Evaporation Physical Vapor Deposition (PVD) in an industrial facility.

### 2.2. Mechanical Characterization

HV_1_ hardness test (load = 1 kgf, dwell time = 10 s) was performed according to ASTM E92-17 [38] to check the T5-C and T6R-C substrate hardness after the deposition cycle. Tensile tests were carried out at room temperature on round dog-bone specimens (Figure 3a) using a screw-testing machine at a strain rate of 3.3 × 10^−3^ s^−1^ according to ISO 6892-1:2020 [39]. YS, UTS, and elongation to failure (e_f_) were evaluated as the average of at least four samples for each investigated condition.

Rotating-bending fatigue tests (R = −1) were performed according to ISO 1143:2021 [40] on hourglass specimens (Figure 3b) by using a single-point rotating-bending machine. The staircase method, defined by ISO 12107:2012 [41], was used to evaluate the fatigue strength at a 50% probability of failure (σ_fs_) and the standard deviation of the fatigue strength distribution. In total, 15 samples for each staircase were tested at 33 Hz, setting a run-out equal to 2 × 10^6^ cycles and a step size of 10 MPa.

### 2.3. Microstructural and Fractographical Analysis

Microstructural analysis was performed by using MIRA3 FEG-SEM (TESCAN, Brno, Czech Republic) with Energy-Dispersive X-ray Spectroscopy (EDS, Brucker Quantax 200/30 mm^2^, Billerica, MA, USA) on cross sections extracted from specimens. Metallographic samples were embedded in conductive resin, ground, polished with diamond suspensions up to 1 µm, according to ASTM E3-11(2017) [42], and finally etched with Weck’s reagent (3 g NH_4_HF_2_, 4 mL HCl, 100 mL H_2_O), according to ASTM E407-07(2015) [43]. At the same time, fractographic analyses were carried out to assess the coating and substrate failure mechanisms by using a multi-focus 3D digital microscope (HIROX, Tokyo, Japan) and the MIRA3 FEG-SEM.

### 2.4. Nanoindentation Tests

The hardness (H) and elastic modulus (E) of Ni-P interlayer and DLC top-coating were analyzed by conducting nanoindentation tests carried out through a NanoTest Vantage (Micromaterials Ltd., Wrexham, UK) equipped with a Berkovich indenter (Centerline-to-face angle, α = 65,27°, Young’s module, E_o_ = 1141 GPa, Poisson’s ratio, ν_o_ = 0.07). Nanoindentation tests were performed with 1 mN/s of load speed, 20 mN as the maximum load, and 5 s holding time at peak load. The mean values were extrapolated from nanoindentation maps of 40 equally spaced indentation points in 25 µm steps. Poisson’s ratios of 0.3 [44] and 0.25 [45] were used to evaluate the E values of the DLC film and the Ni-P coating, respectively. H is evaluated as the ratio between the maximum applied load (P_max_) and the projected contact area at that load (A(h_c_)) (Equation (1)), according to Oliver-Pharr’s method [46].

(1) $$H=\frac{P_{max}}{A(h_{c})}$$

## 3. Results

### 3.1. Microstructural and Nanomechanical Characterization

**Figure 4:**
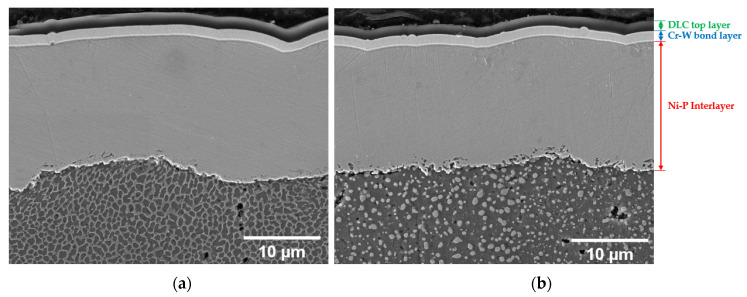
High magnification of the surface in the cross section of the T5-C (**a**) and T6R-C (**b**) samples.

Figure 4 shows the architecture of the multilayer coating deposited onto the PBF-LB AlSi10Mg substrate, where the interlayer Ni-P coating guarantees the good adhesion of the top coating (DLC) to the substrate (PBF-LB AlSi10Mg alloy). An EDS analysis reveals an average P content in the Ni-P interlayer equal to 9.3 ± 0.1 wt%, in agreement with the supplier’s nominal value of 9 wt%.

The Ni-P coating (thickness: 16.5 ± 1.5 µm) shows good adhesion to the Al-Si substrate in both of the heat-treated conditions, characterized by aggregated (T5-C samples) and dispersed Si phases (T6R-C samples) into the Al matrix, (Figure 4), in agreement with [47,48].

**Figure 5:**
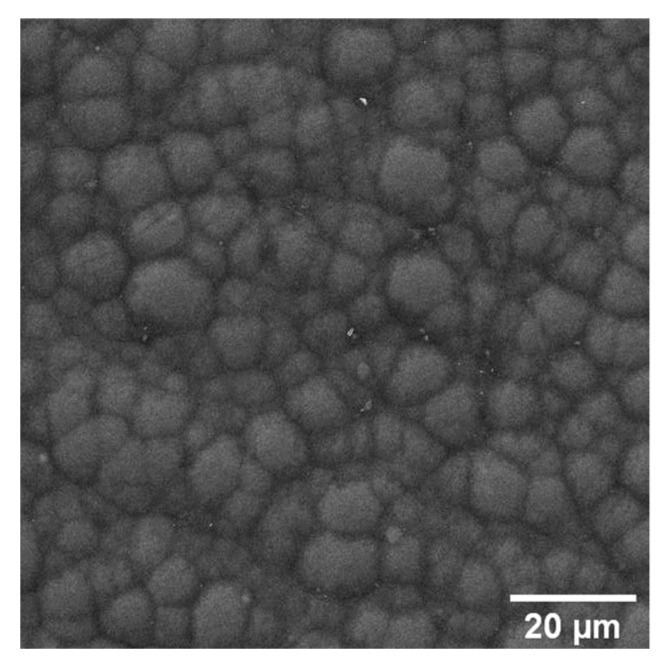
Top view of the multilayer (Ni-P + DLC) coating morphology.

A Cr-W-based bond layer (1.5 ± 0.1 µm) is deposited onto the Ni-P interlayer to improve the adhesion of the DLC top coating (1.3 ± 0.1 µm). Figure 5 shows that the Cr-W bond layers firmly adhere to the Ni-P interlayer, thus replicating its cauliflower surface morphology.

**Figure 6:**
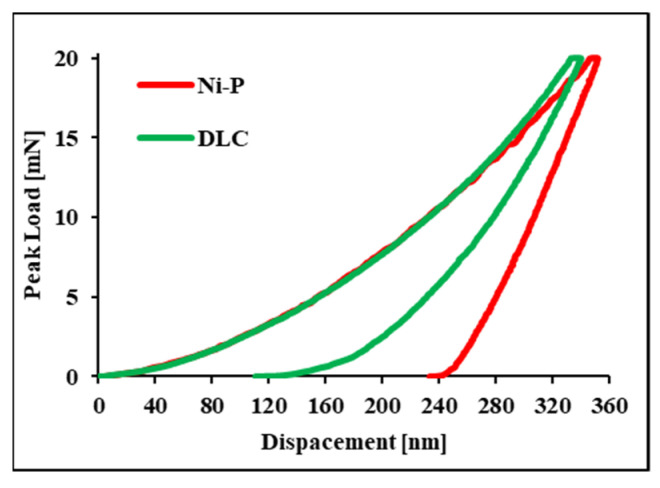
Load-displacement curve of Ni-P + DLC coating measured by instrumented indentation.

Based on loading/unloading curves obtained by nanoindentation tests (Figure 6), the average H and E values of Ni-P are 7.8 ± 0.2 GPa and 130 ± 3 GPa, respectively. These values are comparable to the literature data [10,12,49] and confirm the superior properties compared with the substrate. DLC coating shows average H and E values of 13.3 ± 3 GPa and 129 ± 19 GPa, respectively. In addition to increasing the hardness of the surface, DLC also reduces the coefficient of friction, as described in [23,27,30], promoting its wide use in applications characterized by severe tribological contacts.

Comparable E values characterize the Ni-P and DLC coatings. Having comparable values is an important factor to consider; in fact, as reported by Bouaziz et al. [22], when contiguous layers in a multilayer coating have similar Young’s modulus values, they behave as a single material (assuming perfect adhesion at the interface), avoiding the excessive shear stress at the coating-substrate interface that may cause their detachment.

### 3.2. The Effect of the Multilayer Deposition on the Substrate Microstructure

**Figure 7:**
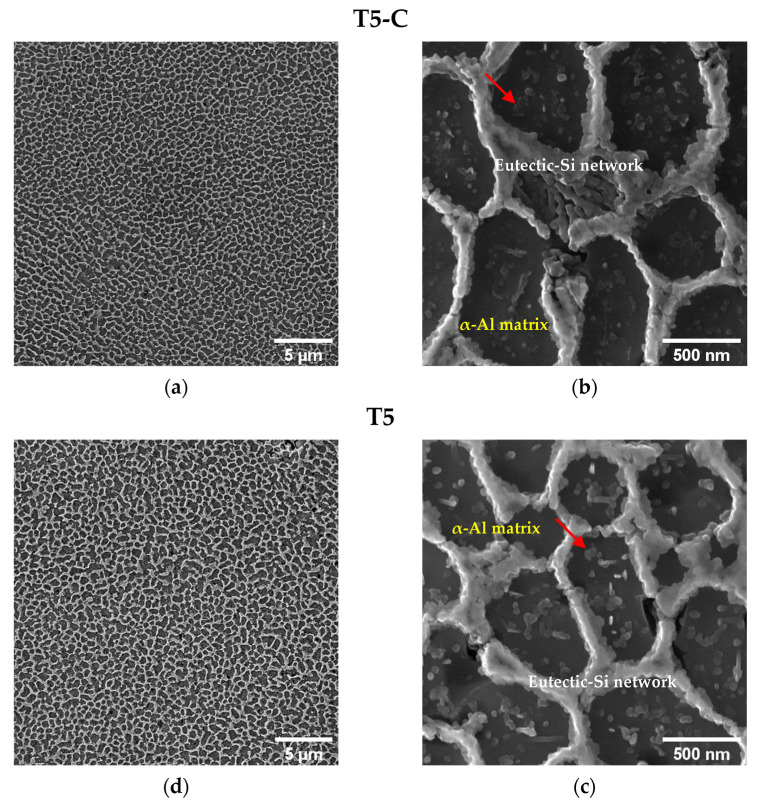
Detail of T5-C (**a**,**b**) and T5 (**c**,**d**) microstructures. The analyzed conditions show a comparable coherent and contiguous eutectic-Si network and a slight difference in the size and density of the Si particles (highlighted by red arrow).

**Figure 8:**
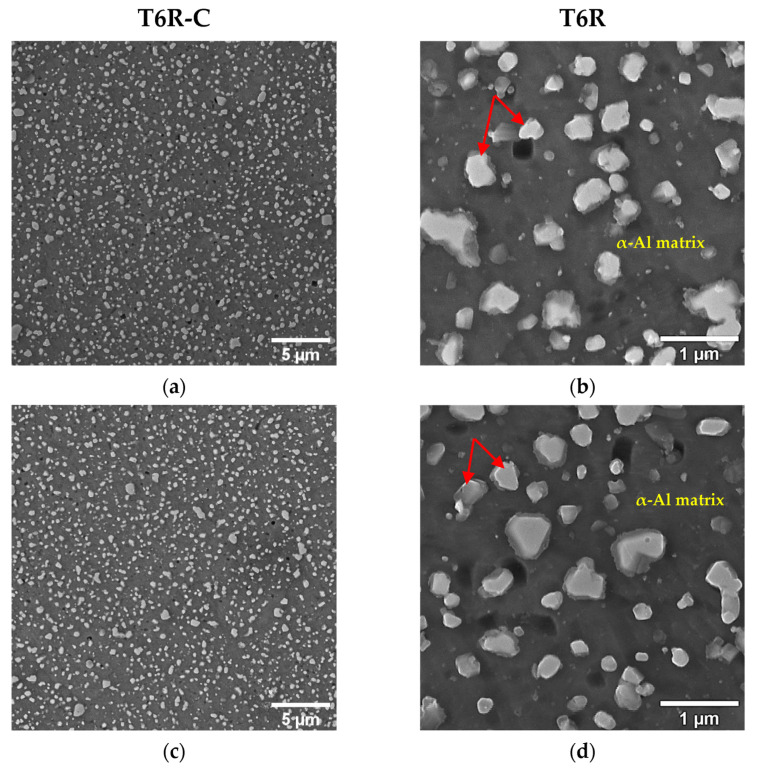
Detail of the PBF-LB AlSi10Mg microstructure after T6R-C (**a**,**b**) and optimized T6R (**c**,**d**). No significant differences in Si particle size or morphology (highlighted by red arrows) were observed.

In Figure 7 and Figure 8, the microstructures of the T5-C and T6R-C specimens are compared with the microstructures of specimens that underwent the optimized T5 (4 h at 160 °C) and T6R (SHTR followed by 6 h at 160 °C) heat treatments described in [33].

The T5-C and T6R-C substrates (Figure 7 and Figure 8) show different microstructural features: (i) branched eutectic-Si network surrounding the submicrometric α-Al cells (Figure 7a,b) and (ii) spheroidal Si particles homogenously distributed in the Al matrix (Figure 8a,b), respectively. The multilayer coating deposition conditions do not promote any remarkable modification of the Si-rich phase in the T5-C and T6R-C compared with the T5 and T6R samples (Figure 7c,d and Figure 8c,d). Only a slight increase in the size of nanometer Si particles and a reduction in number is observed in the T5-C samples (Figure 7d) compared with the optimized T5. Therefore, the coarsening of strengthening precipitates (nanosized Si particles and β-Mg_2_Si precursor phases) induced by diffusion phenomena (Ostwald ripening mechanism) during the DLC deposition can lower the hardness of the T5-C compared with the T5 alloy [3]. As the thermal exposure increases (temperature or soaking time), the strengthening precipitates coalesce, offering less resistance to the dislocation motion. In particular, the formation of larger but fewer precipitates contributes to a lower precipitate-matrix interface area, a higher incoherence with the α-Al matrix, and a lower density of reinforcing phases, thus reducing the hardness of the alloy.

### 3.3. Hardness and Tensile Testing

#### 3.3.1. Mechanical Properties

**Figure 9:**
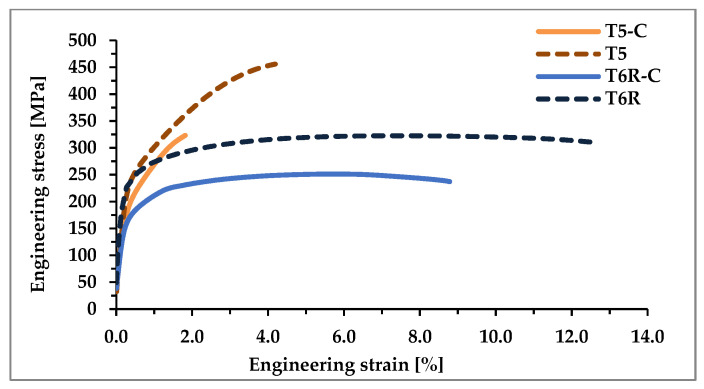
Representative engineering stress-strain curves of the coated (T5-C and T6R-C) and optimized (T5 and T6R) conditions.

Hardness and tensile behavior were evaluated on coated (T5-C and T6R-C) samples and compared with the data reported in [33] referring to T5 and T6R alloy in optimized heat-treatment conditions (T5: 4 h at 160 °C, T6R: 10 min at 510 °C, water quenching, 6 h at 160 °C) (Table 2). Representative engineering stress-strain curves are reported in Figure 9.

The average substrate hardness values of the tensile and fatigue specimens were respectively 122 ± 8 HV_1_ and 85 ± 6 HV_1_ for T5-C and T6R-C. These values are lower than those reported in [33] for specimens that underwent optimized heat treatments: 141 HV_1_ ± 2 and 112 HV_1_ for T5 and T6R specimens, respectively.

The integrated cycle significantly reduces the tensile properties of the heat-treated PBF-LB AlSi10Mg alloy: YS decreases by about 15% and 28% for T5-C and T6R-C, respectively, while UTS by about 29% and 20%, respectively. These data confirm the results of the microstructural analyses, highlighting the effect of nanometric Si precipitate coarsening during the DLC deposition. The higher temperature of DLC deposition compared to the optimal aging temperature (180 °C vs. 160 °C) promotes a significant overaging and the consequent loss in the efficiency of precipitation hardening. Instead, because of the small Ni-P thickness (about 20 µm), the effect of the coating on YS can be considered negligible, not contributing to reducing the detrimental overaging effect [17].

The multilayer coating negatively affects the e_f_ of the samples: the Ni-P interlayer has an amorphous structure characterized by limited ductility (e_f_ value of 1–1.5%), which can sustain limited plastic deformation [21,22]. Therefore, during tensile loading, the crack starts at the Ni-P-Al interface [19] and then propagates in the substrate during plastic deformation, as highlighted by the fractographic analysis. This mechanism leads to a premature failure of the coated samples; in particular, e_f_ values decrease by 58% for T5-C and 31% for T6R-C compared with the T5 and T6R samples, respectively.

These results agree with [3] and confirm that the T5 microstructure undergoes a minor decrease in mechanical properties after thermal exposure compared with the T6R thanks to the higher efficiency of the submicrometric cellular structure in hindering the dislocation motion, thus maintaining significant strength properties.

#### 3.3.2. Fractographic Analysis

**Figure 10:**
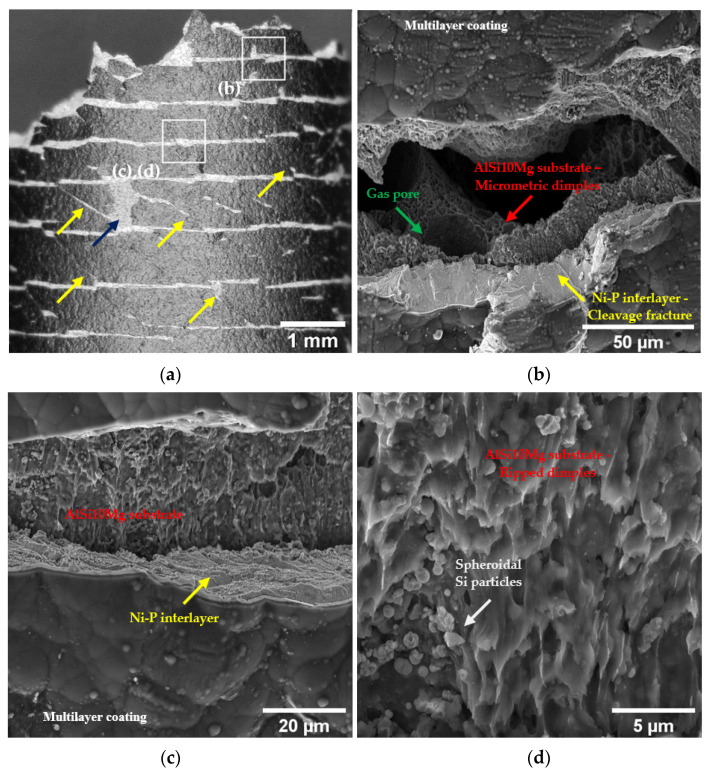
Overview of the cracked coating close to the fracture surface of the T6R-C sample (**a**), with white squares indicating the location of the other images in this figure (**b**–**d**): the blue arrow indicates a coating spalling area; yellow arrows highlight inclined cracks corresponding to maximum shear stress planes. Higher magnification images showing the different fracture mechanisms in Al substrate (ductile) and Ni-P interlayer (brittle) (**b**); Ni-P interlayer delamination from the Al substrate (**c**); surface morphology characterized by ripped dimples and detached Si particles (**d**).

The coated surface of the T6R-C sample shows a high density of cracks and spalling (Figure 10). After exceeding the YS value, the multilayer coating undergoes delamination and fracture. The multilayer acts as a highly brittle material and, once cracked under loading, transfers the energy to the Al substrate thanks to the good adhesion of the Ni-P interlayer (Figure 4), leading to a fast sample failure. The multilayer coating is heavily cracked and extensively delaminated from the surface, and a dense shear-band activity, characterized by very large shear offsets, is observable on the fracture surface (Figure 10a). In particular, the multilayer coating shows open cracks characterized by flat faces and brittle fracture morphology, as described in [22]. In contrast, the substrate is characterized by a ductile fracture morphology, with sub-superficial dimples of different sizes (Figure 10b) and superficial ripped dimples formed by the detachment of the Ni-P interlayer from the substrate (Figure 10d).

**Figure 11:**
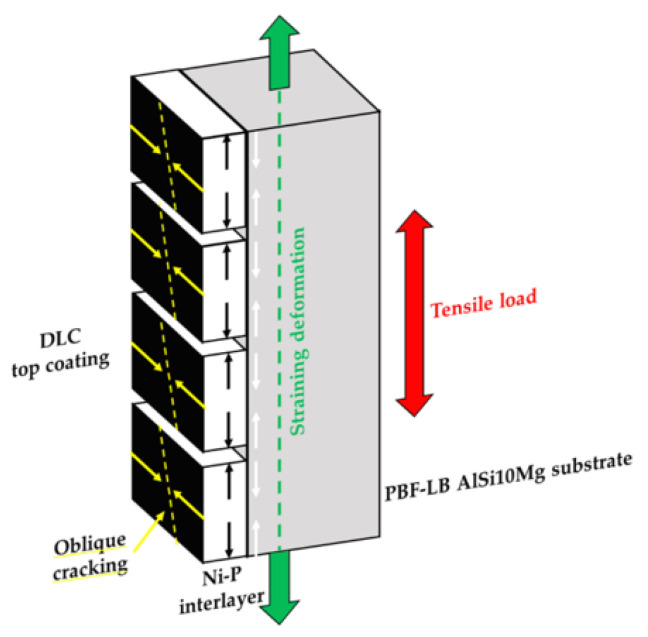
Schematic side view representation of multiple cracking in the coating-substrate system loaded in tensile stress. Debonding mechanisms on the coating result from the compressive/tensile stress condition at the Ni-P coating-substrate interface. Circumferential cracks, perpendicular to the load direction, form at a high strain level, while oblique cracks are associated with the shear stresses generated by substrate necking.

During tensile loading, the different E and υ values between the Ni-P interlayer and the Al substrate promote an overall stress condition consisting of compressive-circumferential and axial stresses that coexist in the coating. At the same time, a compressive axial stress condition is localized in the region immediately below the substrate-coating interface. When the coating fails, the substrate previously in a compressive state suddenly comes into a tensile state, thus easing the fracture initiation [22] (Figure 11). This stress condition leads to the circumferential cracks of the coating and partial debonding from the Al substrate (Figure 10a,c).

At the same time, the maximum resultant shear stress transferred from the substrate to the coating promotes the formation of surface cracks, inclined approximately 45° to the tensile axis (Figure 10a) [19]. The cracks then propagate inward, causing further breakage of the coating. Therefore, Ni-P cracking and debonding are attributed to the maximum resultant shear stress transferred from the substrate to the coating, which causes the failure of the sample and generates inclined cracks (Figure 10a). Ni-P coating cracks are flat thanks to the brittle fracture mechanisms; conversely, the PBF-LB AlSi10Mg alloy has a rough surface associated with high shear stresses at the interfaces, which tear off the topmost portion of the substrate, forming ripped dimples (Figure 10d).

**Figure 12:**
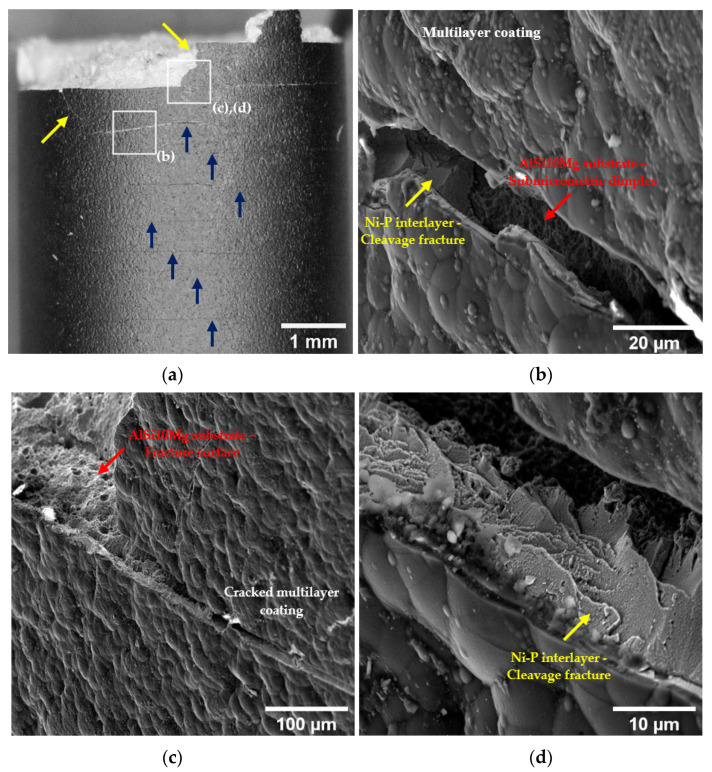
Surface morphology of the multilayer coating close to the fracture surface of the T5-C sample (**a**) with white squares indicating the location of the other images in this figure (**b**–**d**). Yellow arrows point out inclined cracks corresponding to maximum shear stress planes; blue arrows indicate circumferential cracks. Cracking of the multilayer coating close to (**b**) and on (**c**) the fracture surface. High magnification of the cleavage fracture in the Ni-P interlayer (**d**).

Instead, the surfaces of the T5-C samples show few cracks in the coating and are only close to the fracture surface, where strain and stress are more intense (Figure 12). This fracture mechanism is probably linked to the cellular microstructure. This microstructure suffers severe damage in the eutectic-Si network at a low strain because the Si phase is interconnected and cannot accommodate high strain before failure [50,51]. Therefore, even though the causes of coating cracking are the same (excessive stress accumulated in the coating), when the coating begins to fail and the load is transferred to the substrate, the lower strain accommodation capability of the eutectic-Si network leads to the debonding of the coating and the failure of the substrate at the substrate-coating interface.

**Figure 13:**
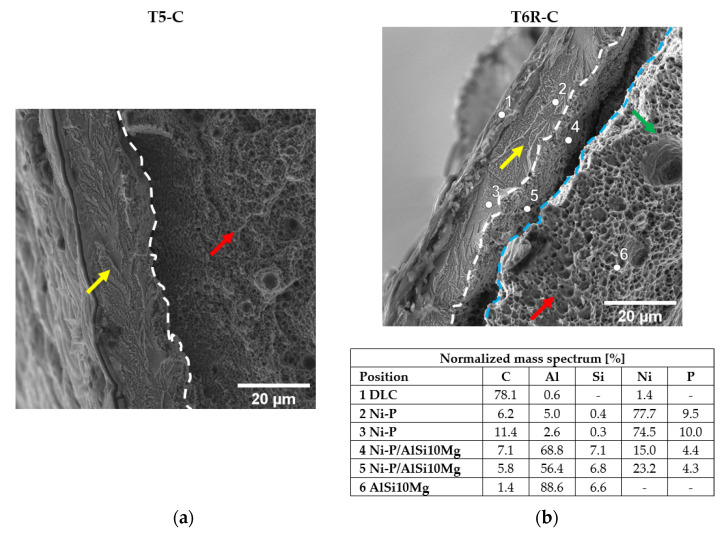
High magnification of the fracture surface at the Al substrate-Ni-P coating interface of the T5-C (**a**) and T6R-C (**b**) samples. In the T5-C sample, the substrate is entirely detached from the coating, while in the T6R-C sample, the detachment is partial. EDS analysis clearly shows that the substrate is still bonded to the Ni-P interlayer. Yellow arrows indicate chevron markings in the Ni-P interlayer, typical of a brittle fracture. Red arrows indicate submicrometric (**a**) and micrometric (**b**) dimples. The green arrow highlights a gas pore in the micrometric dimples (**b**). Dashed white lines indicate the position of the Ni-P-substrate interface (**a**,**b**). Dashed cyan lines indicate the position of the substrate bulk material.

Overall, the Ni-P coating shows strong adhesion to the substrates, but the different microstructures reveal two debonding mechanisms in the T5-C and T6R-C samples (Figure 13). In the T5-C samples, the fibrous and capillary aggregated eutectic-Si network reduces the effective Ni-P-Al matrix interface area, and hence the adhesion of Ni-P interlayer, forming initial sub-superficial cracks and leading to the partial detachment of the substrate from the bulk material during loading application (Figure 13a). In the T6R-C samples, the contact area between the coating and the Al matrix is larger due to the spheroidal morphology of the Si particles, thus improving both the adhesion and the ability of the coating to follow the plastic deformation of the substrate (Figure 13b). Therefore, the fracture occurs within the substrate, a few microns underneath the Ni-P-substrate interface, instead of at the interface. Figure 13b clearly shows the substrate material still bonded to the Ni-P interlayer.

**Figure 14:**
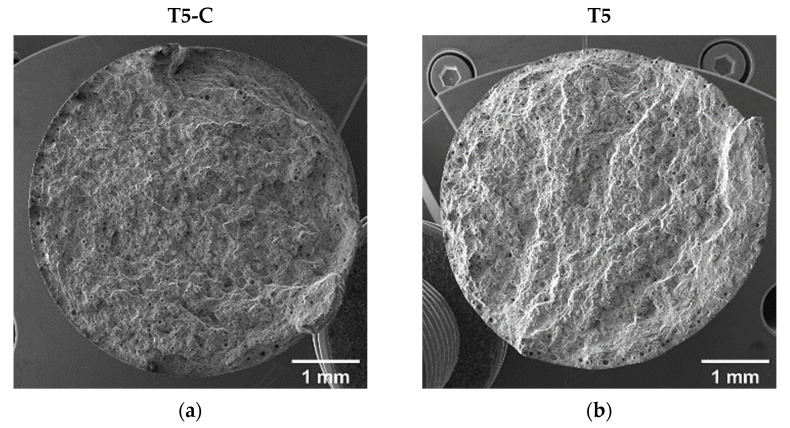

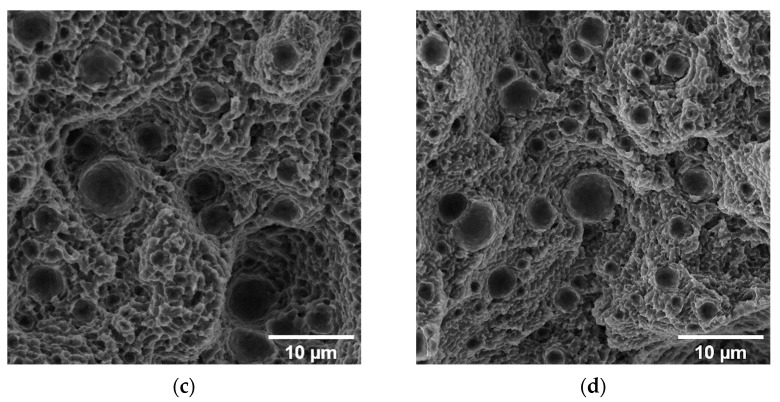
Fracture surfaces at different magnifications of T5-C (**a**,**b**) and T5 (**c**,**d**).

Even though the coated and uncoated samples are characterized by different e_f_, the fracture surfaces show comparable morphologies. The T5-C and T5 samples show step-like features due to interlayer crack propagation (Figure 14a,b). Furthermore, at a higher magnification (Figure 14c,d), the fracture surfaces show shallow dimples induced by plastic deformation associated with the detachment of Al cells from the edges of the eutectic-Si network.

**Figure 15:**
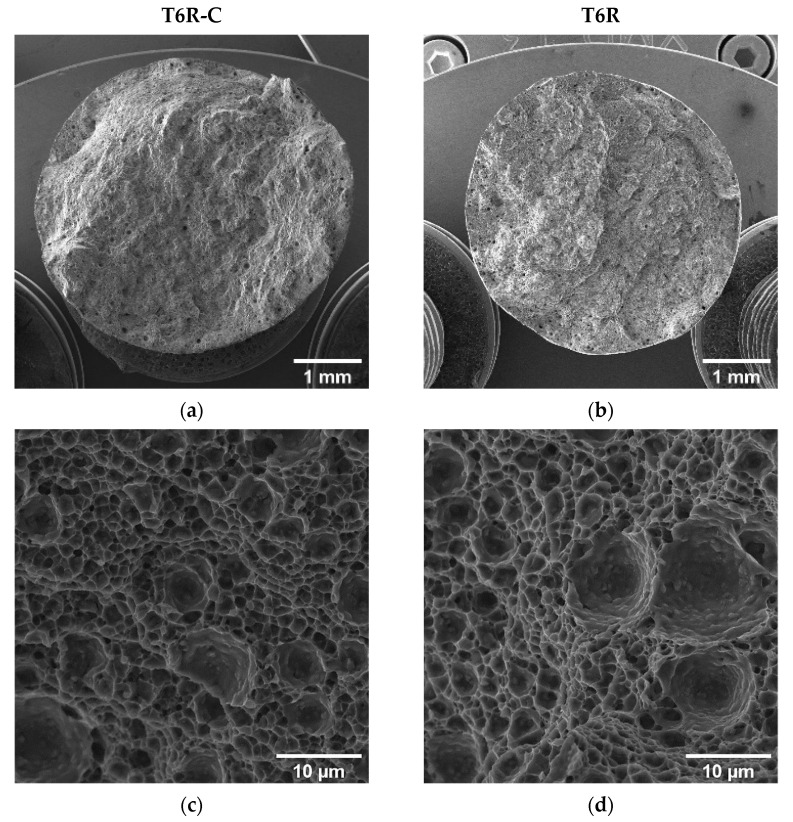
Fracture surfaces of T6R-C (**a**,**c**) and T6R (**b**,**d**): low-magnification (**a**,**b**) and high-magnification (**c**,**d**) images.

As for the T5 and T5-C samples, despite the higher e_f_ of the T6R samples compared with the T6R-C samples, the analyses did not highlight appreciable differences in fracture morphology (Figure 15a,b). The fracture surfaces of the samples are characterized by deep dimples induced by the plastic deformation of the Al matrix around the Si particles (Figure 15c,d), which is typical of ductile failure.

### 3.4. Fatigue Testing

#### 3.4.1. Mechanical Properties

**Figure 16:**
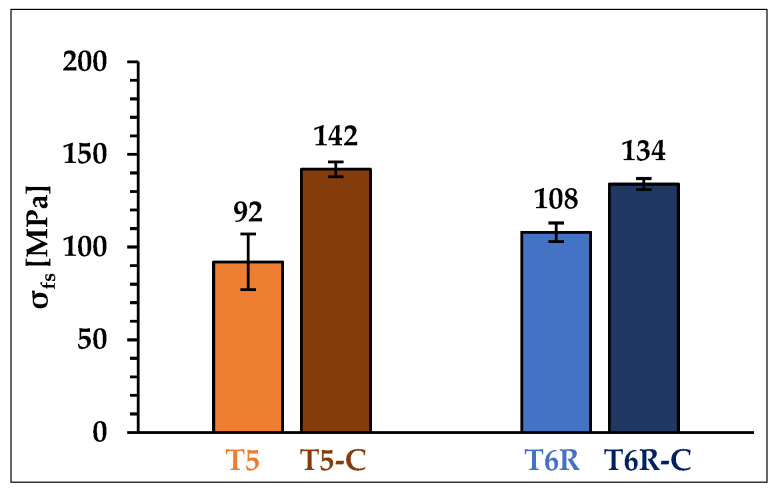
Fatigue strength (2 × 10^6^ cycles) of the heat-treated alloy in optimized (T5 and T6R) and coated (T5-C and T6R-C) conditions. T-bars represent the standard deviation of fatigue strength.

The fatigue test results (Figure 16) indicate an increased fatigue life for the coated PBF-LB AlSi10Mg alloy for both T5-C (+54%) and T6R-C (+24%). Given the decrease in tensile properties thanks to the thermal load applied during DLC topcoat deposition and the close correlation between UTS and fatigue strength in Al alloys [52,53], this result appears very interesting.

Probably, the higher strength of the Ni-P interlayer than the substrate and the development of compressive residual stresses during deposition [16] can delay the crack initiation at the surface or subsurface of the specimens [13], reducing the detrimental influence of the surface or subsurface defects on crack initiation. A further consequence of the decrease in the deleterious effects of the defects on crack initiation is the better fatigue behavior of the T5-C samples compared to T6R-C samples due to the higher strength of its peculiar cellular microstructure.

#### 3.4.2. Fracture Surface Analysis

**Figure 17:**
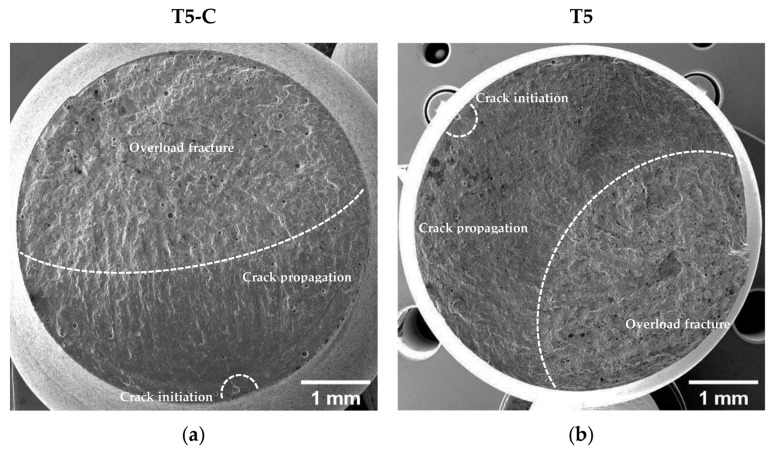

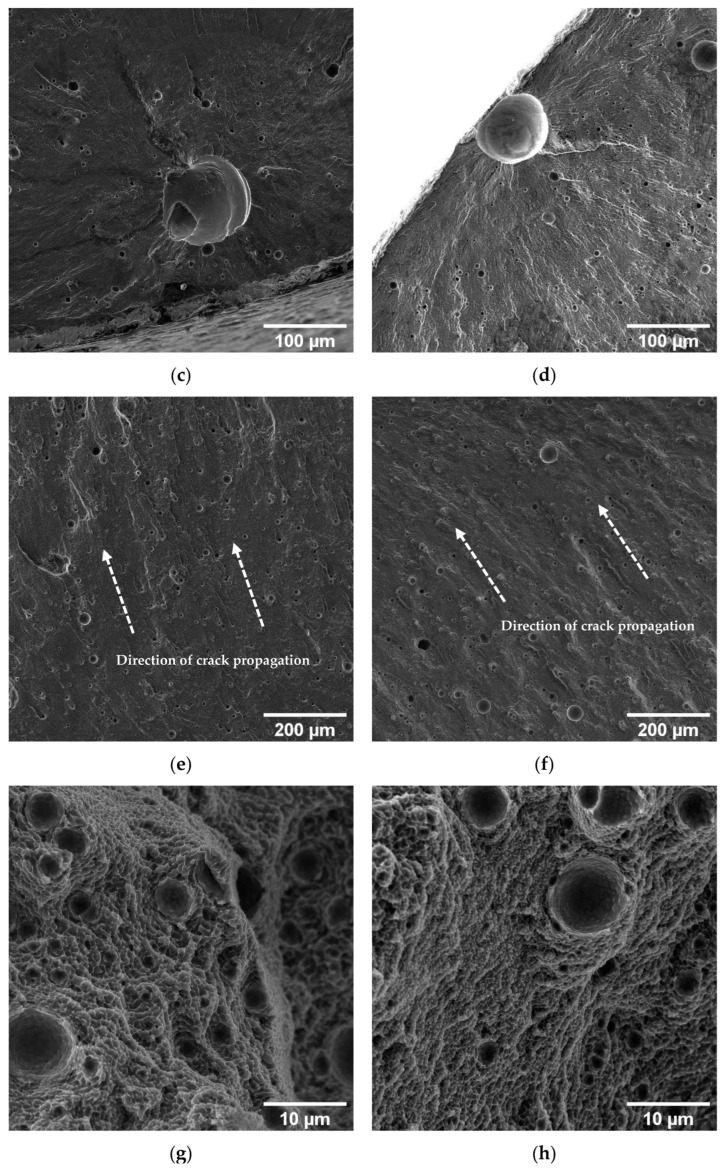
SE image of the T5-C (**a**,**c**,**e**,**g**) and T5 (**b**,**d**,**f**,**h**) samples. Fracture surface (**a**,**b**); crack initiation (**c**,**d**); crack propagation region (**e**,**f**); micrometric dimple voids in the overload fracture area (**g**,**h**). Dotted white arrows indicate the direction of crack propagation.

**Figure 18:**
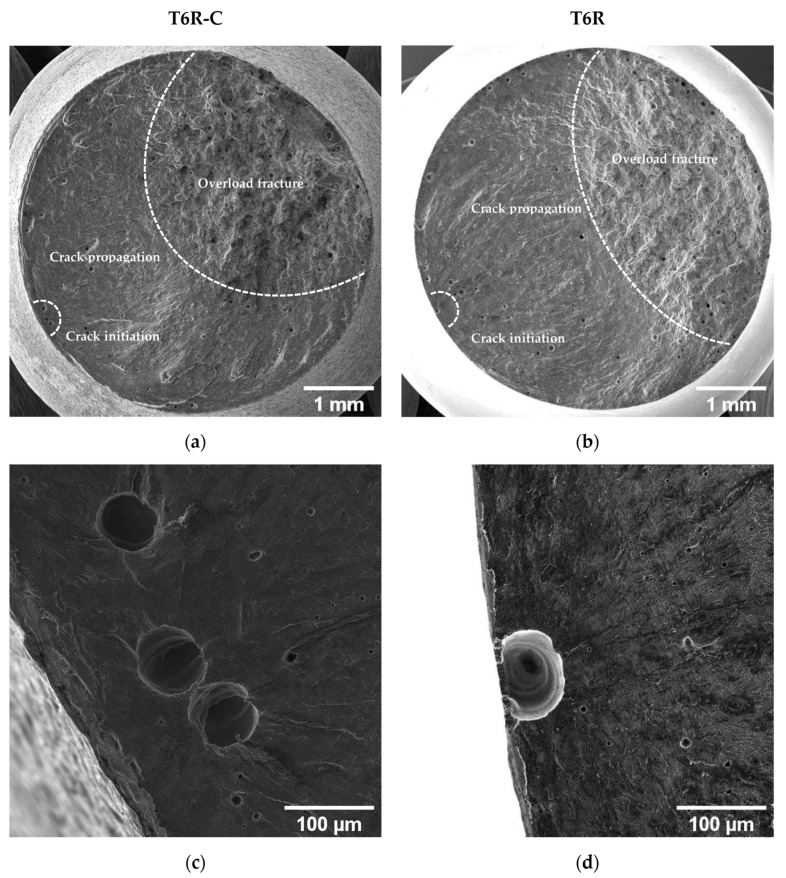

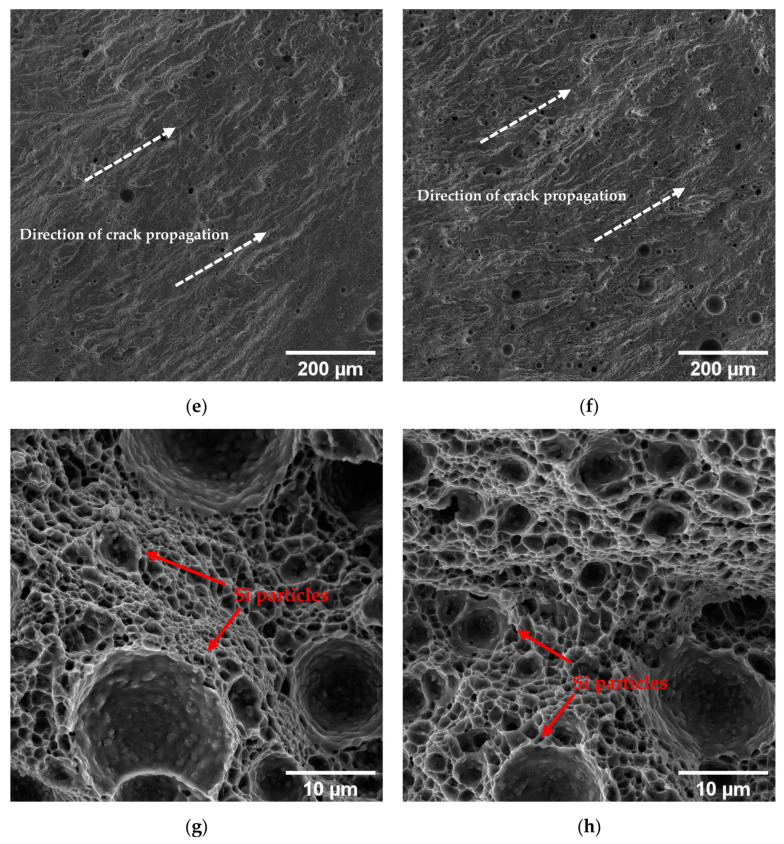
SE image of the T6R-C (**a**,**c**,**e**,**g**) and T6R (**b**,**d**,**f**,**h**) samples. Fracture surface (**a**,**b**); crack initiation (**c**,**d**); crack propagation region (**e**,**f**); micrometric dimple voids in the overload fracture area (**g**,**h**). Red arrows show the Si particles inside large dimples.

Figure 17 and Figure 18 compare the fatigue fracture surface of all the analyzed conditions (T5C, T5, T6R-C, and T6R). An overall observation of the surface (Figure 17a,b and Figure 18a,b) shows comparable failure mechanisms, consisting of (i) crack initiation, (ii) propagation, (iii) and final overload fracture.

In all the failed samples, the convergence of the radial fracture lines reveals crack initiation: a large pre-existing pore, characterized by a diameter between 50 and 100 µm, located along the circumference of the specimen (Figure 17c,d and Figure 18c,d), which introduces high-stress concentration. However, in uncoated samples, cracks nucleate near the surface (Figure 17d and Figure 18d), while in DLC-coated samples (Figure 17c and Figure 18c), crack initiation is located in a sub-superficial position at a depth ranging from 100 to 200 µm. In particular, the superior coating hardness, the high Ni-P interlayer adhesion to the substrate, and the compressive residual stresses introduced by the coating deposition process prevent the initiation of cracks from the coating or the substrate-coating interface, as observed in other studies [13,17,18,26]. This hypothesis is supported by the fact that at the crack initiation zone, the coating shows a completely brittle fracture without secondary cracks or features typical of fatigue failure (Figure 17c and Figure 18c).

The Ni-P coating does not modify the fracture mechanisms in the PBF-LB AlSi10Mg alloy, which is dominated by single crack propagation (Figure 17e,f and Figure 18e,f). Therefore, the fatigue crack surface shows a planar and stable crack propagation characterized by micro-tearing and fatigue striations (Figure 17e,f and Figure 18e,f) in the fatigue propagation region, which radiate away from the initiation sites and follow the crack-growth direction.

The overload fracture zone exhibits ductile behavior, as described for tensile specimens. In particular, T5-C and T5 (Figure 17g,h) show shallow dimples caused by the plastic deformation of the α-Al cells and the detachment along the border of the eutectic-Si network, showing small tear-ridge facets and dense shear ridges mixed with dimple regions, typical of a quasi-brittle fracture. In the T6R-C and T6R samples, the final fracture area displays a ductile behavior with relatively deep dimples, compared to the T5 specimens, thanks to plastic relieving and larger gas pores caused by the SHTR (Figure 18g,h).

In short, significant differences exist between the results of fatigue tests carried out on samples that were heat treated according to the optimal parameters (T5 and T6R) and the samples that underwent the integrated cycles of coating and heat treatment (T5-C and T6R-C). In particular, coating deposition induces a significant increase in fatigue strength. As Murakami et al. [54] described, the size and morphology of the defects and their distance from the surface influence the fatigue strength of the material. Therefore, introducing the multilayer coating reduces sensitivity to the defect by moving the possible crack initiation zone away from the surface and increasing specimen resistance to the crack opening through increased surface hardness and residual compressive stresses in the coating and substrate.

This mechanism is more effective on the T5-C alloy, where the deposition of the multilayer coating limits the effects of the more-stress-sensitive T5 microstructure to crack initiation so that the higher strength properties of the substrate (UTS and hardness) compared with those of T6R lead to higher fatigue strength, overturning the results obtained in the uncoated, polished conditions (T5 and T6R).

## 4. Conclusions

This work investigated the mechanical performance of the PBF-LB AlSi10Mg alloy coated with the Ni-9%P + DLC (a-C:H) multilayer coating. In particular, two integrated deposition and heat-treatment cycles were developed, where the DLC deposition substituted artificial aging (AA) in the T5 and T6R heat treatments. The first cycle consists of Ni-P + DLC deposition on the as-built alloy (T5-C) and the second of Ni-P + DLC deposition on the alloy after the rapid solution (SHTR) at 510 °C for 10 min (T6R-C). Microstructural and mechanical characterizations were carried out (tensile and rotating fatigue tests) to evaluate the influence of the multilayer coating and integrated cycles on mechanical performance. Fracture surfaces were analyzed to identify substrate and multilayer coating damage mechanisms and possible effects on fatigue failure. The results were compared with the data from previous research on uncoated samples subjected to optimized T5 (AA at 160 °C for 4 h) and T6R (SHTR at 510 °C for 10 min, followed by AA at 160 °C for 6 h) heat treatments. The following conclusions can be drawn:

- Integrating the heat-treatment cycle into the multilayer coating deposition process does not induce appreciable modifications in the morphology or dimensions of the hard Si-rich phase of the eutectic network and the secondary spheroidal Si phase in the T5-C and T6R-C microstructures.
- The DLC coating deposition conditions promote significant overaging of the substrate, which leads to a decrease in the YS and UTS values compared with optimized conditions (T5 and T6R): −15% and 29% for T5-C and −28% and 31% for T6R-C, respectively.
- Differences in elastic-plastic properties between the multilayer coating and the PBF-LB AlSi10Mg substrate lead to cracking at the Ni-P-substrate interface and propagation in the substrate during plastic deformation. However, the homogeneous distribution of spheroidal Si particles in the T6R microstructure increases the adhesion and the ability of the coating to follow the plastic deformation of the substrate compared with the T5 microstructure, leading to a lower loss in terms of e_f_ (−58% and −31%, respectively).
- Extensive coating cracking and spalling occur during the tensile tests thanks to the complex compressive/tensile stress condition at the Ni-P coating-substrate interface. Circumferential cracks, perpendicular to the load direction, form at high strain levels, while oblique cracks are associated with the shear stresses generated by substrate necking.
- Multilayer Ni-P + DLC coating increases the fatigue strength of the T5 alloy (+54%) and the T6R alloy (+24%) thanks to the residual compressive stresses in the coating and the substrate. Moreover, the coating remains well adherent to the substrate during fatigue testing, not becoming a source of fatigue cracks.
- The multilayer coating does not modify the main fracture mechanisms of the substrate in tensile and fatigue specimens.

Even though the thermal exposure during DLC deposition induces a significant overaging of the alloy, thus reducing the tensile properties, the fatigue performance significantly improves, outlining the possible application of an integrated cycle consisting of heat treatment and coating deposition for high-performance engineering components operating in severe stress conditions. Last but not least, integrating the coating and heat-treatment cycles allows for reducing post-processing times and costs.

## Figures and Tables

**Table 1:** Process parameters used for the production of the specimens.

Laser Power [W]	Scan Speed [mm/s]	Spot Diameter [μm]	Layer Thickness [μm]	Hatch Distance [μm]
350	1150	80	50	170

**Table 2:** Tensile properties and hardness of the PBF-LB AlSi10Mg alloy related to the following conditions: (i) T5-C, (ii) T5 (AA at 160 °C for 4 h), (iii) T6R-C, and (iv) T6R (SHTR at 510 °C for 10 min followed by AA at 160 °C for 6 h).

	YS [MPa]	UTS [MPa]	e_f_ [%]	HV_1_ (Substrate)
T5-C	217 ± 1	323 ± 4	1.8 ± 0.0	122 ± 8
T5	256 ± 3	452 ± 3	4.3 ± 0.6	141 ± 2
T6R-C	180 ± 5	252 ± 4	8.7 ± 0.5	85 ± 6
T6R	251 ± 4	319 ± 6	12.6 ± 0.7	112 ± 1

## Data Availability

The raw/processed data required to reproduce these findings cannot be shared at this time, because the data also form part of an ongoing study.
